# Extended-spectrum beta-lactamase *Escherichia coli* and *Klebsiella pneumoniae* urinary tract infections

**DOI:** 10.1017/S0950268820003015

**Published:** 2020-12-17

**Authors:** P. Vachvanichsanong, E. B. McNeil, P. Dissaneewate

**Affiliations:** 1 Department of Pediatrics, Prince of Songkla University, Hat Yai, Songkhla, Thailand; 2 Epidemiology Unit, Faculty of Medicine, Prince of Songkla University, Hat Yai, Songkhla, Thailand

**Keywords:** Congenital anomalies of the kidney and urinary tract, extended-spectrum beta-lactamase *Escherichia coli*, *Klebsiella pneumoniae*, recurrent urinary tract infection

## Abstract

The prevalence of extended**-**spectrum beta**-**lactamase (ESBL)-producing *Escherichia coli* and *Klebsiella pneumoniae* urinary tract infections (UTIs) is increasing worldwide. We investigated the prevalence, clinical findings, impact and risk factors of ESBL *E. coli*/*K. pneumoniae* UTI through a retrospective review of the medical records of children with UTI aged <15 years admitted to Prince of Songkla University Hospital, Thailand over 10 years (2004–2013). Thirty-seven boys and 46 girls had ESBL-positive isolates in 102 UTI episodes, compared with 85 boys and 103 girls with non-ESBL isolates in 222 UTI episodes. The age of presentation and gender were not significantly different between the two groups. The prevalence of ESBL rose between 2004 and 2008 before plateauing at around 30–40% per year, with a significant difference between first and recurrent UTI episodes of 27.3% and 46.5%, respectively (*P* = 0.003). Fever prior to UTI diagnosis was found in 78.4% of episodes in the non-ESBL group and 61.8% of episodes in the ESBL group (*P* = 0.003). Multivariate analysis indicated that children without fever (odds ratio (OR) 2.14, 95% confidence interval (CI) 1.23–3.74) and those with recurrent UTI (OR 2.67, 95% CI 1.37–5.19) were more likely to yield ESBL on culture. Congenital anomalies of the kidney and urinary tract were not linked to the presence of ESBL UTI. In conclusion, ESBL producers represented one-third of *E. coli*/*K. pneumoniae* UTI episodes but neither clinical condition nor imaging studies were predictive of ESBL infections. Recurrent UTI was the sole independent risk factor identified.

## Introduction

*Escherichia coli* and *Klebsiella pneumoniae* are the two most common causative organisms of urinary tract infection (UTI) in childhood [1, 2]. Both species frequently produce extended-spectrum beta-lactamase (ESBL) enzymes which confer resistance to beta-lactam antibiotics including third- and fourth-generation cephalosporins, and monobactams [3, 4].

The increasing isolation of ESBL-producing *E. coli* and *K. pneumoniae* causing UTI in children is of concern worldwide due to the failure of empirical therapy which may result in serious clinical complications such as sepsis, renal scarring and prolonged hospitalisation, compared to infection with non-ESBL strains [2–11].

The isolation of ESBL-positive organisms limits therapeutic options and these patients invariably require parenteral antibiotic therapy. Ideally, appropriate empirical antibiotics should be prescribed immediately on the presentation of a suspected UTI before susceptibility results are available but most empirical regimens lack activity against ESBL-positive isolates. As a consequence, the majority of ESBL diagnoses and treatments are often delayed subject to antimicrobial susceptibility data.

Given the current increasing rates of antibiotic resistant uropathogens worldwide, it is highly necessary to monitor and evaluate the characteristics and outcomes of UTI, particularly in children, to facilitate early diagnosis and initiate appropriate treatment to minimise potentially hazardous sequelae. Additionally, the impact of ESBL UTI on antibiotic therapy needs to be determined as well as its association with congenital anomalies of the kidney and urinary tract (CAKUT). Knowledge of differences in clinical presentations, urine findings and kidney and urinary tract imaging results between ESBL and non-ESBL UTI in children will potentially inform timely antibiotic selection prior to the results of urine cultures are known.

## Methods

We retrospectively reviewed the medical records of children diagnosed with both nosocomial and community-acquired UTI aged <15 years admitted to Prince of Songkla University Hospital in southern Thailand from January 2004 to December 2013. Only children yielding a pure culture of *E. coli* or *K. pneumoniae* were included in the study. UTI was confirmed by urine culture as growth of ≥5 × 10^4^ cfu /mm^3^ for catheter specimens and ≥1 ×  10^5^ for clean catch midstream urine. Urine cultures and antimicrobial susceptibility testing using a standard agar disk diffusion method with 15 antimicrobial agents were performed by the hospital laboratory.

Pyuria was defined as >10 neutrophils per high-power field, and proteinuria was detected using a positive dipstick test (Beckman Coulter Ireland Inc., Co., Clare, Ireland) or a quantitative urine protein to creatinine ratio of >0.2 mg/mg. An abnormally high creatinine value was defined as any level above the upper normal range for age [12].

The results of renal ultrasounds (RUS), voiding cystourethrograms (VCUGs) and ^99m^Tc dimercaptosuccinic acid (DMSA) renal scans were obtained from routine reports. Hydronephrosis was determined by measuring the anteroposterior mid-renal pelvic diameter and graded as mild, moderate or severe according to the system used by the Society for Fetal Urology [13]. VCUG was performed to determine the anatomy of the lower urinary tract system, including the urinary bladder and urethra, particularly for diagnosis of vesicoureteral reflux, ureterocele and/or posterior urethral valve. DMSA renal scans were performed according to the standard clinical procedure and at least 5 months after a confirmed UTI was evaluated. A normal DMSA scan is defined as no focal defect uptake of the tracer with an uptake difference between left and right kidneys of <10%. An abnormal DMSA scan indicated either renal scarring or dysplasia. Renal scarring is defined as a diffuse or sharp indentation in the kidney contour with thinning of the cortex. Dysplasia is defined as a differential renal function <35% [14].

The prevalence of ESBL-causing UTI, clinical findings, laboratory results and imaging studies were compared between the two groups of ESBL and non-ESBL UTI children.

### Statistical analysis

The prevalence of UTI caused by ESBL and non-ESBL *E. coli* and *K. pneumoniae* was compared using the *χ*^2^ test. Descriptive statistics are presented using medians and interquartile ranges (IQR) for continuous variables, and frequency and percentage for categorical variables. Comparisons of the proportions of ESBL *vs.* non-ESBL UTI episodes across demographic and clinical factors were made using the rank-sum test or *χ*^2^ test as appropriate. Independent risk factors of ESBL UTI were determined using multivariate logistic regression models. Statistical significance was considered as a *P*-value <0.05. R version 3.6.0 was used for all analyses [15].

## Results

### Demographic characteristics

Of 336 children diagnosed with 429 UTI episodes during the study period, *E. coli* and *K. pneumoniae* were causative in 271 children (80.7%) and 324 episodes (75.5%). The median (IQR) age of the children at these episodes was 1.4 (0.6–4.1) years. Among the 271 children, 83 (37 boys, 46 girls) had 102 episodes in which at least one of their urine culture confirmed as an ESBL strain, and 188 children (85 boys, 103 girls) had 222 episodes in which all of the urine cultures were non-ESBL. Table 1 shows a comparison of the demographic and clinical factors between the ESBL and non-ESBL UTI episodes. The median (IQR) ages at presentation were 1.5 (0.7–4.8) years for the ESBL group and 1.3 (0.6–3.9) years for the non-ESBL group (*P* = 0.2). The proportions of ESBL infections among boys and girls were 30.3% and 32.6%, respectively (*P* = 0.7).

**Table 1.** Comparison of demographic and clinical factors between ESBL and non-ESBL among 324 UTI episodes

	Total (*N* = 324)	ESBL (*n* = 102)	Non-ESBL (*n* = 222)	*P*-value
Age, median (IQR), months		1.5 (0.7–4.8)	1.3 (0.6–3.9)	0.2
Age group				0.9
≤6 months	58	17 (29.3)	41 (70.7)	
6–12 months	73	20 (27.4)	53 (72.6)	
1–2 years	53	17 (32.1)	36 (67.9)	
2–5 years	71	24 (33.8)	47 (66.2)	
6–15 years	69	24 (34.8)	45 (65.2)	
Sex				0.7
Male	152	46 (30.3)	106 (69.7)	
Female	172	56 (32.6)	116 (67.4)	
UTI episode				0.003
First	253	69 (27.3)	184 (72.7)	
Recurrent	71	33 (46.5)	38 (53.5)	
Fever				0.003
Febrile	237	63 (26.6)	174 (73.4)	
Afebrile	87	39 (44.8)	48 (55.2)	
Pyuria				0.07
Pyuria	295	88 (29.8)	207 (70.2)	
No pyuria	29	14 (48.3)	15 (51.7)	
Proteinuria				0.07
Proteinuria	97	38 (39.2)	59 (60.8)	
No proteinuria	226	64 (28.3)	162 (71.7)	
Serum creatinine				<0.001
Abnormally high	42	10 (23.8)	32 (76.2)	
Normal	226	81 (35.8)	145 (64.2)	
Not measured	56	11 (19.6)	45 (80.4)	

ESBL, extended-spectrum beta-lactamase; IQR, interquartile range; UTI, urinary tract infection.

### Clinical findings and laboratory results

The prevalence of ESBL in first UTI episodes was 27.3% compared to 46.5% in recurrent episodes (*P* = 0.003) and among those with fever prior to UTI diagnosis was 26.6% compared to 44.8% of those without fever (*P* = 0.003). Fever was more common in younger (age <2 years) children (79.5%) compared to older (age >2 years) children (64.7%) (*P* = 0.003). The prevalence of ESBL-positive isolates among children with pyuria and proteinuria was 48.3% and 39.2%, respectively, compared to 29.8% and 28.3% among those without these conditions (both *P* = 0.07). Interestingly, the prevalence of ESBL in children with an abnormally high creatinine level was lower than for those with normal creatinine values (23.8% and 35.8%, respectively; *P* = 0.2).

### Changes in ESBL prevalence over time

**Fig. 1.**
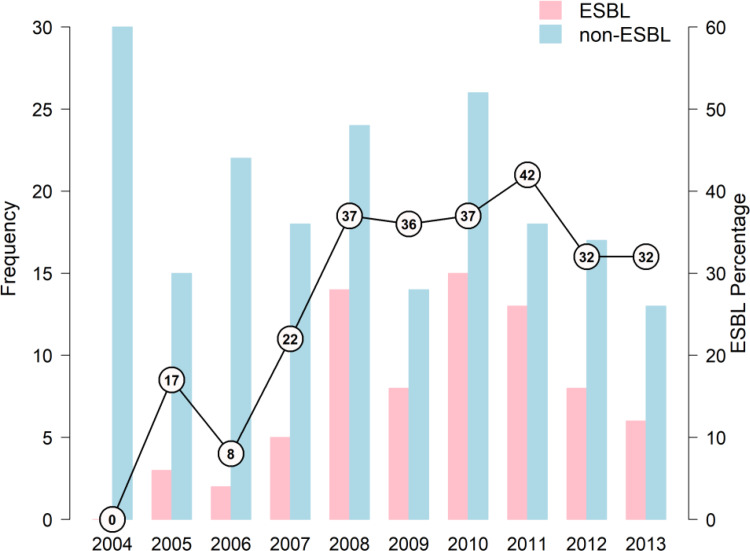
Number of children with *E. coli*/*K. pneumoniae* ESBL- and non-ESBL-caused UTIs per year. The line represents the percentage of ESBL-caused UTIs each year. ESBL, extended*-*spectrum beta*-*lactamase.

Figure 1 shows the prevalence and proportion of ESBL and non-ESBL UTI for each year of the study. No ESBL-producing strain of the target species was found at the start of the study in 2004 but by 2008, this phenotype accounted for over 30% of isolates and remained stable thereafter at around 30–40% per year. ESBL *E. coli* was more common than ESBL *K. pneumoniae* but ESBL production among *E. coli* strains (70/265 = 26.4%) was lower than among *K. pneumoniae* strains (32/59 = 54.2%, *P* < 0.001).

### Antibiotics

**Fig. 2.**
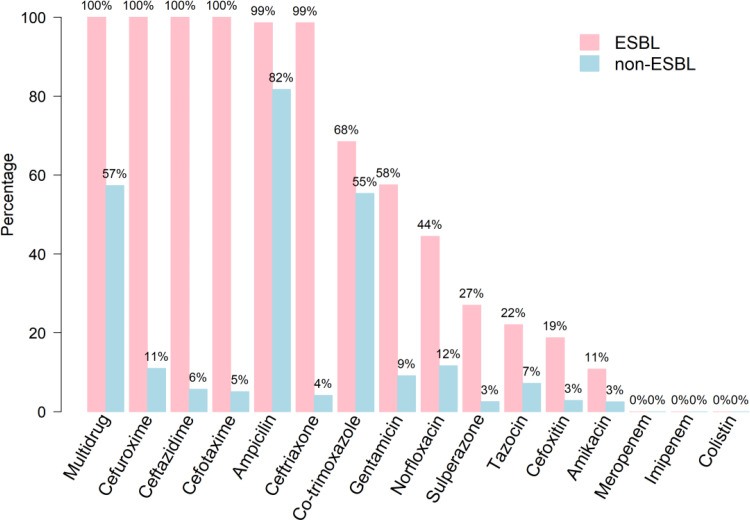
Antibiotic resistance of bacterial isolates stratified by ESBL and non-ESBL UTIs.

The resistance rates of isolates to the antimicrobials tested stratified by ESBL and non-ESBL are shown in Figure 2. All ESBL isolates showed uniform resistance to cefuroxime, ceftazidime and cefotaxime and 99% were resistant to ampicillin and ceftriaxone. Overall, non-ESBL isolates were markedly more susceptible to the majority of the antimicrobials tested with the exception of ampicillin (83% resistant) and co-trimoxazole (58%); <13% of this group showed resistance to all other antimicrobials. Multi-drug resistance – defined as resistance to three or more antibiotic classes – was uniform for ESBL isolates compared with 59% of non-ESBL. Regardless of ESBL status, all isolates proved susceptible to meropenem, imipenem and colistin.

Ceftriaxone was the most common antibiotic empirically prescribed (69.8%) followed by ertapenem (7.4%) and ceftazidime (6.8%). There was no difference in clinical condition and responsiveness between patients with ESBL UTI who were started on antibiotics which were not effective against ESBL organisms and who subsequently received appropriate antibiotics for such organisms, and those patients who were initially treated appropriately with anti-ESBL agents.

### Imaging study results

A comparison of imaging study results and kidney abnormality findings between children with ESBL and non-ESBL UTI (Table 2) showed that the former group had abnormal RUS, VCUG and DMSA scans (36.1%, 46.8% and 42.2%, respectively), compared to those with normal imaging results (20.8%, 19.2% and 17.6%; *P* = 0.018, *P* < 0.001 and *P* = 0.016, respectively).

**Table 2.** Comparison of imaging study results and kidney abnormality findings between 271 children with ESBL- and non-ESBL-caused UTI^a^

	Total (*N* = 271)	ESBL (*n* = 83)	Non-ESBL (*n* = 188)	*P*-value
Imaging study				
RUS	227 (83.8%)			0.018
Normal	144	30 (20.8)	114 (79.2)	
Abnormal	83	30 (36.1)	53 (63.9)	
VCUG	192 (70.8%)			<0.001
Normal	130	25 (19.2)	105 (80.8)	
Abnormal	62	29 (46.8)	33 (53.2)	
DMSA renal scan	96 (35.4%)			0.015
Normal	51	9 (17.6)	42 (82.4)	
Abnormal	45	19 (42.2)	26 (57.8)	
Any detected CAKUT	129 (47.6%)	45 (34.9)	84 (65.1)	<0.001
Primary vesicoureteral reflux	52	22 (42.3)	30 (57.7)	
Isolated hydronephrosis	43	8 (18.6)	35 (81.4)	
Neurogenic bladder	18	10 (55.6)	8 (44.4)	
Isolated renal damage	6	0 (0.0)	6 (100.0)	
Ureteropelvic junction obstruction	3	2 (66.7)	1 (33.3)	
Posterior urethral valve	3	2 (66.7)	1 (33.3)	
Duplex kidney	2	1 (50.0)	1 (50.0)	
Other^b^	2	1 (50.0)	1 (50.0)	
No detected CAKUT	142 (52.4%)	38 (26.8)	104 (73.2)	<0.001

CAKUT, congenital anomalies of the kidney and urinary tract; DMSA, ^99m^Tc dimercaptosuccinic acid; ESBL, extended-spectrum beta-lactamase.

a ESBL-caused UTI indicates at least one culture testing positive for ESBL.

b Vesicorectal fistula, urethrovaginal fistula.

Of the 271 children, 233 had at least one imaging study performed to diagnose CAKUT; the remaining 38 children with no imaging study were significantly older (median age = 5.1 years) compared to those with CAKUT (median age = 1.3 years) and those with no CAKUT (median age = 11 months). CAKUTs were detected in 129 (47.6%) children, of which 34.9% had ESBL UTI, while the prevalence of ESBL UTI among those without CAKUT was 26.8% (*P* = 0.19). The most common congenital anomaly was primary vesicoureteral reflux (VUR) (*n* = 52), followed by isolated hydronephrosis (*n* = 43) and neurogenic bladder (*n* = 18). Six children were found to have isolated renal damage without other kidney or bladder anomalies and all had non-ESBL UTI.

Table 3 shows the results of the multivariate analysis predicting ESBL *E. coli*/*K. pneumoniae*-caused UTI. The significant predictors were the lack of fever and recurrent UTI. Children with an unknown CAKUT status were four times more likely to have ESBL UTI than those with no CAKUT based on imaging studies but there was no difference in the frequency of ESBL UTI between children with CAKUT compared to those without. Afebrile children with UTI (odds ratio (OR) 2.14) and those with a recurrent UTI (OR 2.67) were significantly more likely to have an ESBL producer as a causative agent.

**Table 3.** Multivariate results predicting ESBL-caused UTIs among 324 episodes

	Crude OR (95% CI)	Adjusted OR (95% CI)	*P*-value (Wald's test)	*P*-value (LR-test)
Age group (ref. <6 months)				0.291
6–12 months	0.86 (0.40–1.83)	0.81 (0.36–1.82)	0.607	
1–2 years	1.08 (0.48–2.39)	0.74 (0.30–1.81)	0.507	
2–5 years	1.16 (0.55–2.44)	0.44 (0.18–1.08)	0.073	
>5 years	1.16 (0.55–2.46)	0.43 (0.17–1.06)	0.068	
Fever (ref. febrile)				0.007
Afebrile	2.24 (1.35–3.74)	2.14 (1.23–3.74)	0.007	
Creatinine (ref. normal)				0.072
High	0.56 (0.26–1.20)	0.48 (0.21–1.08)	0.076	
Not measured	0.44 (0.21–0.89)	0.52 (0.24–1.14)	0.103	
Pyuria (ref. no pyuria)				0.246
Pyuria	0.46 (0.21–0.98)	0.59 (0.24–1.43)	0.243	
Proteinuria (ref. no proteinuria)				0.140
Proteinuria	0.61 (0.37–1.00)	0.65 (0.37–1.15)	0.138	
UTI episode (ref. 1st episode)				0.003
Recurrent UTI	2.32 (1.35–3.98)	2.67 (1.37–5.19)	0.004	
CAKUT (ref. no CAKUT)				0.007
CAKUT	2.08 (1.18–3.66)	1.70 (0.89–3.21)	0.105	
Unknown	4.23 (2.00–8.94)	4.05 (1.67–9.79)	0.002	

CAKUT, congenital anomalies of the kidney and urinary tract; CI, confidence interval; LR, Likelihood ratio; ref, reference group; OR, odds ratio.

## Discussion

Our study in this group of Thai children with UTI showed that the prevalence of ESBL-producing *E. coli*/*K. pneumoniae* increased in the decade between 2004 and 2013 but has remained steady at around 30–40% to the time of this study in 2020 (unpublished data). The proportion of *K. pneumoniae* ESBL was approximately double that of *E. coli* ESBL. Neither age, gender, pyuria, proteinuria, abnormally high creatinine nor the presence of CAKUT was predictive of an ESBL strain as a cause of UTI. However, a previous history of UTI and being afebrile prior to presentation were significantly more common in the ESBL group. Older children had a significantly lower prevalence of fever (*P* = 0.003), and although those with pre-diagnosis fever had a significantly lower prevalence of ESBL, age was not associated with ESBL.

The country-specific prevalence of ESBL *E. coli*/*K. pneumoniae*-caused UTI varies widely but has tended to increase over time in many regions [2–11]. Reported prevalence rates include 43.5% overall from Egypt (41.9% *E. coli* and 48.8% *K. pneumoniae*) [16], and similarly in Cambodia [2]. Likewise, in Turkish children almost half of 142 cases overall were infected with the ESBL strains of *E. coli* or *K. pneumoniae*, with the latter species accounting for over 60% of cases [11]. Two studies reporting ESBL UTI rates in children in India range from 21.7% to 33.2%, with a higher prevalence in females in one [10], and 37.5% in another [17]; these rates are comparable to those found in the current study. In contrast, rates of around 20% have been reported from more recent studies in Thailand and Korea [9, 18]. In one study in infants, age <1 year was reported to be an independent risk factor for ESBL UTI (OR 1.73, 95% confidence interval (CI) 1.08–2.78), however the 95% CI was very close to 1 [19].

Fever, a non-specific symptom, is an important sign of UTI in infants, and together with pyuria leads to a urine culture being performed. However, somewhat surprisingly, pre-diagnosis fever was more prevalent in our non-ESBL UTI children group. For afebrile children, a delayed diagnosis of UTI is unavoidable. In our study, the proportion of children aged >2 years was 43.2%, and as noted, UTI in older children had a lower prevalence of fever than in infants <2 years. Overall, 87 (26.9%) UTI episodes had no fever. Even though ESBL was more common in afebrile children, this symptom has limited application in clinical practice because there is no rationale for expecting or considering a diagnosis of UTI.

Recurrent UTI episodes were more likely to be caused by ESBL-producing strains than first UTI episodes (OR 2.7), a result consistent with two other studies from Turkey and Korea [9, 11]. However, in the Korean study, CAKUT also proved to be an independent risk factor for ESBL-caused UTI, which is in contrast to our findings. Indeed a separate multivariate analysis of our data (not shown due to small sample size) found that previous a ESBL UTI increased the risk of a recurrent infection with these organisms by threefold.

Serum creatinine levels were available for only 268 (83%) of the episodes, but there was no significant difference in the prevalence of abnormally high levels between the ESBL and non-ESBL groups. Likewise, indicators for CAKUT were similarly distributed between the groups, and unlike our previous study where CAKUT was identified as a risk factor for recurrent UTI [20], it was not an independent risk factor for ESBL UTI. Almost half of our UTI patients were aged more than 2 years, which, in our setting, may be due to the fact that parents of children with symptoms of UTI tend to delay seeking medical care for their children. Another possibility is that these parents may seek care for their child at another health facility prior to referral to our tertiary care centre. Children who did not have an imaging study performed were older, possibly because their parents delayed seeking care or the original doctors followed the UTI investigation guidelines from more developed countries which recommend imaging studies as first-line investigations only in infants. Alternatively, parents of older children may be less inclined to return for imaging investigations after their children have been discharged from hospital.

ESBL-caused UTI is serious in terms of antibiotic resistance since patients require parenteral antibiotic therapy and admission to hospital. As a consequence, diagnosis of ESBL UTI and treatment with appropriate antibiotics are usually delayed until the results of urine cultures are known. However, our findings suggest that the more serious consequences of ESBL UTI such as urosepsis, CAKUT association and renal damage, may not be as great a concern as originally thought. Recurrent UTI and being afebrile were the only identified factors associated with ESBL UTI. We suggest that empirical antibiotics which may not be active against ESBL strains should still be considered for use even in recurrent UTI episodes until a urine culture positively indicates ESBL, and thus allow specific therapy to be initiated. Such practice will aid the reduction of drug resistance through a more rational use of antibiotics and a strict antibiotic prescription policy.

Regarding ESBL UTI, the choice of antibiotic is highly important and careful prescription of agents should therefore be made taking into context local species distributions and susceptibility data [21]. Similarly, the duration of treatment should be guided by the need to balance maximum effectiveness of therapy while minimising the opportunity for the development of drug resistance. In our patient cohorts both ESBL and non-ESBL strains were uniformly susceptible to meropenem, imipenem and colistin while >80% of ESBL producers were susceptible to amikacin and cefoxitin. Cefoxitin appears to be a promising agent due to the low resistance rate, but it is not yet recommended for treatment of childhood UTI. Moreover, its use is complicated because minimum inhibitory concentrations need to be monitored continually and doses adjusted accordingly [22], hence the preference for the other four drugs. For non-ESBL-infected children, there are more choices as such organisms in this study were found to have <50% susceptibility to only two drugs (ampicillin and co-trimoxazole). A study from Turkey in 53 children aged 3–12 years with amikacin-sensitive ESBL UTI [23] reported a 96% response rate with once daily intramuscular amikacin for 6 days (range 3–7 days). Overall, 30% of the children had recurrent UTI, a similar proportion was given antibiotic prophylaxis, and the majority was prescribed antibiotics in the ensuing 3 months**.** Amikacin can be used as a second**-**line drug but should be limited to patients with amikacin**-**sensitive pathogens who are not able to be admitted for intravenous antibiotics**.** However, it should be prescribed with caution due to issues of oto**-** and nephrotoxicity, particularly in patients with compromised renal function.

Our study has some limitations. First, it was retrospective and thus histories of previous antibiotic and prophylactic therapy were not available; however, histories of antibiotic therapy in some Thai communities are often unreliable due to the wide availability of antimicrobials from drug stores; and whether the child completes the course of antibiotics is also not known. Second, some children with VUR may have had antibiotic prophylaxis but as there was no association between ESBL and VUR, we presume that antibiotic prophylaxis (co-trimoxazole) was unlikely to be related to ESBL UTI. Finally, the relatively small sample size did not allow sub-group analyses to be made.

Administration of the appropriate antibiotic for ESBL UTI is often compromised due to time taken for urine testing processes and reporting. This delays diagnosis and initiation of treatment and can contribute significant morbidity such as renal scarring, hypertension and renal impairment in worst case scenarios [24, 25]. No such immediate consequences were evident in our study.

In conclusion, approximately one-third of all childhood UTI episodes were due to *E. coli*/*K. pneumoniae* ESBL producing strains. They first appeared in 2003 and increased dramatically over the ensuing 5 years before plateauing at 30–40%. All ESBL strains were susceptible to carbapenems and colistin. Fortunately, ESBL infections did not prove to be any more complicated than non-ESBL cases and did not result in immediate and serious consequences. Neither the clinical condition nor imaging studies of patients were predictive of the presence of ESBL strains, but recurrent UTI and being afebrile were the only two independent patient risk factors identified for the development of ESBL UTI.

## Data Availability

Data and other materials that would be necessary to replicate the findings of this paper are available upon request from the corresponding author.
